# Acetabular Reconstruction with the Burch-Schneider Antiprotrusio Cage and Bulk Allografts: Minimum 10-Year Follow-Up Results

**DOI:** 10.1155/2014/194076

**Published:** 2014-05-21

**Authors:** Dario Regis, Andrea Sandri, Ingrid Bonetti

**Affiliations:** Department of Orthopaedic and Trauma Surgery, Integrated University Hospital, Piazzale A Stefani 1, 37126 Verona, Italy

## Abstract

Reconstruction of severe pelvic bone loss is a challenging problem in hip revision surgery. Between January 1992 and December 2000, 97 hips with periprosthetic osteolysis underwent acetabular revision using bulk allografts and the Burch-Schneider antiprotrusio cage (APC). Twenty-nine patients (32 implants) died for unrelated causes without additional surgery. Sixty-five hips were available for clinical and radiographic assessment at an average follow-up of 14.6 years (range, 10.0 to 18.9 years). There were 16 male and 49 female patients, aged from 29 to 83 (median, 60 years), with Paprosky IIIA (27 cases) and IIIB (38 cases) acetabular bone defects. Nine cages required rerevision because of infection (3), aseptic loosening (5), and flange breakage (1). The average Harris hip score improved from 33.1 points preoperatively to 75.6 points at follow-up (*P* < 0.001). Radiographically, graft incorporation and cage stability were detected in 48 and 52 hips, respectively. The cumulative survival rates at 18.9 years with removal for any reason or X-ray migration of the cage and aseptic or radiographic loosening as the end points were 80.0% and 84.6%, respectively. The use of the Burch-Schneider APC and massive allografts is an effective technique for the reconstructive treatment of extensive acetabular bone loss with long-lasting survival.

## 1. Introduction

Revision of the acetabular component of a total hip arthroplasty (THA) with associated bone loss is a complex challenge, due to the difficulty to obtain a primary stability and to reconstitute periprosthetic bone stock.

Minor, cavitary bone defects can be successfully treated by porous-coated, hemispherical cups [1, 2].

Conversely, the optimal option for management of uncontained deficiencies is still a controversial issue, because stable fixation and long-term bone ingrowth are not reliable.

Filling acetabular bony cavities with massive allografts resulted in early failure due to resorption of the graft [3–7].

Segmental acetabular defects involving both columns with more than 50% of the graft supporting the cup suggest the application of ilioischial devices [8–13].

The antiprotrusio cage (APC) was originally designed by Burch in 1974 and later modified by Schneider in 1975 to manage protrusion acetabuli. The aim was to bridge areas of bone loss, providing immediate mechanical fixation.

In pelvic bone deficiencies, APC proved out to be an effective treatment option with successful mid- to long-term outcomes [14–24].

The aim of this study was to evaluate the minimum 10-year clinical and radiographic outcome of massive allografts combined with the Burch-Schneider antiprotrusio cage for the management of severe combined deficiencies in failed total hip arthroplasty.

## 2. Materials and Methods

Between January 1992 and December 2000, 97 hips with periprosthetic acetabular bone loss in 94 patients were revised using bulk allografts and the Burch-Schneider APC.

Twenty-nine patients, for a total of 32 implants, deceased for unrelated reasons with a well-functioning THA still in place before the minimum 10-year clinical and radiographic assessment was reached.

Sixty-five hips in 65 patients were available for retrospective evaluation at an average of 14.6 years (range, 10.0 to 18.9 years) postoperatively. They were 16 males and 49 females, with a mean age at surgery of 60 years (range, 29 to 83 years). No case was lost to follow-up. The indication for revision surgery was painful aseptic loosening of the cup with extensive acetabular bone loss in 62 hips. Other reasons included second stage surgery following spacer block implantation for infection in 2 cases and periprosthetic femoral fracture associated with cup loosening in 1 case. The femoral component was replaced simultaneously in all but 6 patients.

The Burch-Schneider antiprotrusio cage (Sulzer Orthopedics Ltd., Winterthur, Switzerland) has been made of smooth-blasted titanium up to 1998, when it was manufactured from a biocompatible TiAlNb alloy with a rough-blasted surface. The device consists of an elliptic basket, with a proximal flange fixed to the ilium with multiple screws, and a distal flange which is driven into the ischium. The implant is specific for right and left side and at the time of the operations was available only in two sizes (44 and 50 mm).

The assessment of acetabular bone loss was determined from both preoperative radiographs and intraoperative findings after removal of the socket and documented using the Paprosky et al. classification system [25]. All patients had type III bone defects, indicating severe bone loss with superior migration greater than 3 cm and medial osteolysis. Type IIIA defect was detected in 27 hips and type IIIB in 38 hips, the difference being the integrity or the break of Kohler's line, respectively.

When plain X-ray revealed a massive acetabular bone deficiency with endopelvic protrusion of the cup, a preoperative digital angiography was routinely performed to define vascular location. In the presence of an acetabular component in close proximity to the iliofemoral vessels, revision hip surgery was preceded by a laparotomic approach isolating the iliac vessels, so that major vascular bleeding could be prevented [26]. In the present series, digital angiography and laparotomy were performed in 9 and 7 patients, respectively.

In all cases acetabular defects were filled using structural allografts, which were composed of 1 or more femoral heads deep-frozen at −80°C and sterilized by autoclaving (at that time, fresh-frozen bone was not available). Intraoperatively the graft was reamed and sized to closely press-fit the residual host bone, and pelvic fixation was never supplemented with screws.

The Burch-Schneider cage was shaped to provide optimal congruity to the grafted acetabulum. The superior and inferior flanges were bended in order to comply with the individual anatomy of the acetabular region and to maximize the stability of the cage. The inferior flange was driven into a precut slot in the ischium in 56 cases (86.2%) and buttressed against the ischium in 9 cases (13.8%). Iliac fixation was obtained using 2 to 5 cancellous screws, which were placed first in the acetabular dome and then were transversely positioned through the proximal flange. The polyethylene inner socket was cemented inside the metal cage with an appropriate orientation. Antithromboembolic drugs (low-molecular-weight heparin) and short-term antibiotics were administered routinely. Ambulation was allowed 1 week after surgery, but the patients were requested to restrict load on the revised hip for a minimum of 2 months. Progressive weight bearing with crutches or a walker then started and full weight bearing was achieved 4–6 months after the operation.

Clinical examination included the grading of pain, walking ability, and joint motion according to the Harris hip score (HHS) [27].

Standard anteroposterior and lateral radiographs of the pelvis and the involved hip were obtained preoperatively, immediately after surgery, and on outpatient controls at 6 weeks, 3 months, 6 months, 12 months, and annually thereafter. Radiographic assessment was performed by a single observer.

The stability of the acetabular component was evaluated according to the classification of Gill et al. [28], which differentiates a cage into definitely loose (screw breakage or acetabular migration of 5 mm or progressive radiolucent lines at the cage-bone interface medially and superiorly or around the screws), probably loose (progressive radiolucencies present medially or superiorly), and possibly loose (nonprogressive radiolucent lines and no involvement of the screws). However, van der Linde and Tonino [17] and Symeonides et al. [22] concluded that breakage of the screws without continuing migration or change in orientation of the cage should not be defined as failure. The osseointegration of the structural allograft was assessed on anteroposterior view using Gross criteria [29]. Resorption was graded as minor (<1/3 of graft resorbed), moderate (1/3 to 1/2 of graft resorbed), and severe (>1/2 of graft resorbed). The formation of ectopic ossifications was determined using the classification of Brooker et al. [30].

The cumulative survival rate of the prostheses was estimated according to the method of Kaplan-Meier, using two end points for the entire series of 65 hips: removal for any cause or X-ray migration of the reconstruction cage and mechanical failure including aseptic and radiographic loosening of the metal device. Survivorship analysis was reported with a 95% confidence interval (CI).

The preoperative and follow-up Harris hip scores were compared with use of the Wilcoxon signed-rank test as data were not normally distributed according to skewness-kurtosis test. The level of significance was *P* < 0.01 [31].

## 3. Results

The patients were followed up until the most recent evaluation, cage revision, or death. All unrevised hips (56) were available for clinical and radiographic assessment, with follow-up ranging from 10.0 to  18.9 years (median, 14.6 years).

### 3.1. Reoperations

A total amount of 9 cages (13.8%) required further surgical revision. In 3 hips the Burch-Schneider device was removed because of unresponsive deep infection, which occurred in the early postoperative period. Five patients underwent revision for aseptic loosening and progressive migration of the reconstruction cage between 3 and 12 years after index surgery. Finally, 1 APC was replaced because of breakage observed in a 34-year-old male following massive polyethylene cup wear. In 2 cases rerevision was successfully achieved using cementless porous-coated sockets, while the remaining 4 hips have been operated on with the same reconstructive procedure. No acetabular cage revision was performed in 56 patients.

### 3.2. Complications

Postoperatively, 2 deep vein thromboses occurred with no lasting sequelae. A 71-year-old female patient developed a permanent palsy of the femoral nerve. One patient developed a transient sciatic nerve palsy which fully recovered within six months.

Local complications included 6 early dislocations (9.2%) which were managed successfully by closed reduction and 4 weeks of bracing. No recurrence was observed. As mentioned previously, 3 hips required removal of the prosthesis because of chronic infection.

### 3.3. Clinical Results

The average Harris hip score improved significantly (*P* < 0.001) from 33.1 points (range, 1 to 81) preoperatively to 75.6 points (range, 46 to 97) at the time of the latest examination.

### 3.4. Radiographic Results

A complete roentgenographic assessment was taken for all survived hips (56) at scheduled times according to the study protocol.

In addition to the rerevised cases, 4 acetabular cages were considered definitely loose based on the classification of Gill et al. at mean follow-up of 14.6 years, but the patients refused further surgical treatment because of relatively slight pain and low-physical demand. In these cases, migration of the acetabular component was associated with severe resorption of the original graft, indicating the failure of the reconstructive treatment. Minor or moderate resorption of the allograft was detected as well, both in 2 patients. Forty-eight hips (73.8%) had evidence of full incorporation of the structural graft.

**Figure 1:**
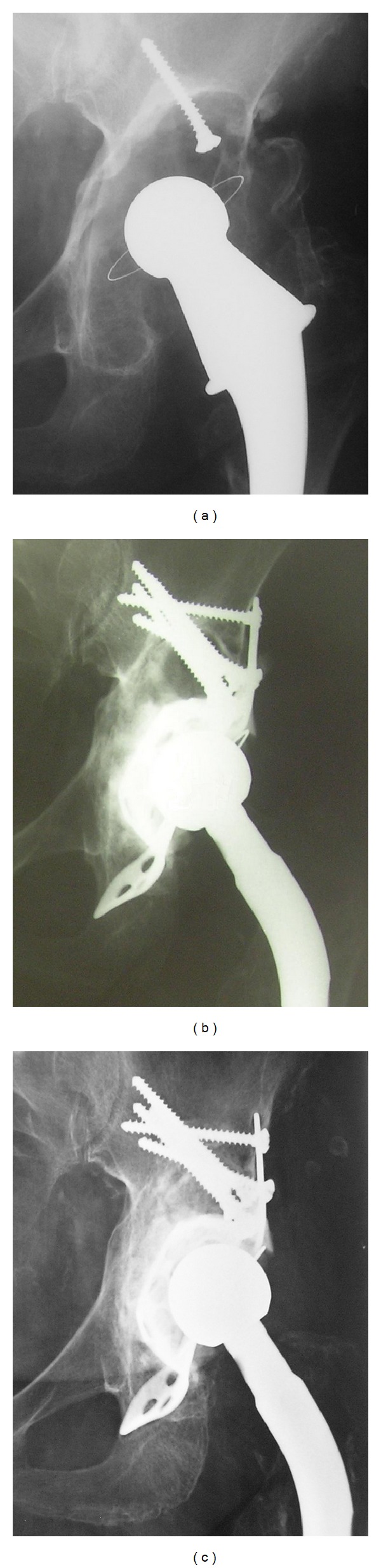
Preoperative X-ray of a 71-year-old female patient with extensive acetabular bone loss (type IIIB) around cemented cup (a). Radiograph after revision with the Burch-Schneider APC and structural allograft (b). Fourteen-year follow-up shows the stability of the reconstruction cage and the incorporation of the bone graft (c).

In 52 cases (80%) X-ray signs of stability of the Burch-Schneider cage were assessed at the time of the latest follow-up (Figure 1).

The development of heterotopic bone formation was detected in 13 hips (23.2%) and was graded as class I and class II in 10 and 3 cases, respectively. The occurrence of periprosthetic ossifications was not responsible for pain or functional impairment.

### 3.5. Survival Analysis

**Figure 2:**
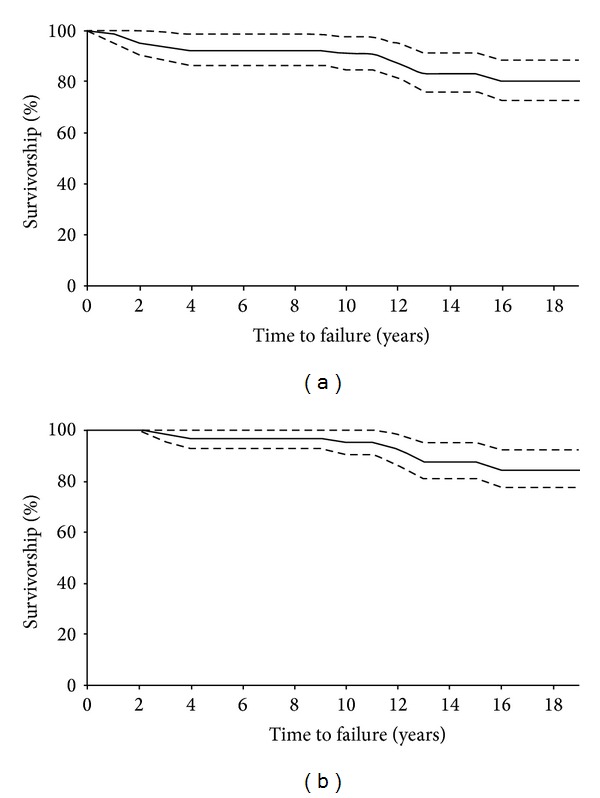
The Kaplan-Meier survivorship curves and 95% confidence intervals for the Burch-Schneider cage and bulk allografts with failure defined as X-ray migration and removal for any cause of the cage (a) and aseptic or radiographic loosening (b).

Nine of 65 reconstructive APCs had to be rerevised (during the follow-up period) and 4 resulted in being radiographically loose. Using the failure definition of removal for any reason or X-ray migration of the reconstruction cage, the cumulative survival rate was 80.0% (95% CI, 72.6%–88.1%) after 18.9 years (Figure 2(a)).

The survivorship of the Burch-Schneider device with removal for aseptic loosening or radiographic failure as the end point was 84.6% at 18.9 years with a 95% confidence interval between 77.5% and 92.4% (Figure 2(b)).

## 4. Discussion 

The management of severe deficiency of pelvic bone stock in revision hip surgery is a critical problem because of the difficulty in providing a stable and durable fixation of the new prosthesis. Various treatment options have been described for reconstructing the mechanically compromised acetabular columns, including the use of cementless jumbo or oblong cups, reinforcement rings, antiprotrusio cages, ilioischial cages with modular trabecular metal augments, structural and morselized bone grafting.

Conventional cementless cups with supplementary screw fixation have shown excellent long-term results with survival rates of 95% after 15 years in contained acetabular revisions [1, 2].

However, biologic fixation of porous-coated sockets is unlikely when there is less than 50% contact with living host bone.

Several reports have demonstrated that filling the defective bone with unsupported massive periacetabular allografts has resulted in early failure because of graft resorption and cup loosening [3–7].

When the graft supports more than 50% of the acetabular component, a reconstruction system spanning bone defects from ilium to ischium should be used to protect the graft and provide structural stability [5, 10–13, 32].

The rates of success of cage reconstruction have been conflicting, ranging from 69% to 100%. Comparison of results of cages is difficult because of the mixed patient populations treated with different devices and the variable acetabular bone loss [8, 14, 17, 33–41].

The Burch-Schneider antiprotrusio cage is designed to manage extended pelvic defects by bridging large bone gaps and protecting the grafts filled to increase the bone stock. Mechanical failure rate between 0% and 15% and radiographic loosening from 0% to 24% have been reported at midterm follow-up [15, 16, 18, 19, 29, 36, 42–45], but most series do not collect selective results of using cages only for the most severe defects [8, 9, 20, 22, 28, 33, 36, 46–49]. Probably, the varying results are related also to surgical technique [11] and different types of bone grafts used to fill pelvic deficiency [41].

The first large series (42 cases) of reconstructions using the Burch-Schneider cage in revision arthroplasty was reported by Berry and Müller in 1992 [42]. The high rate of aseptic loosening (12%) after an average follow-up of 4.7 years (with 5 additional septic failures) was related to the use of morselized bone graft. Subsequently, this surgical technique has been used by several authors with highly variable outcome. Rosson and Schatzker found no revisions and 5 nonprogressive radiolucencies in 20 hips at a mean of 6 years [33]. Significant component migration was documented by Peters et al. in 14% of 28 reconstructions at an average of 33 months postoperatively [50]. Wachtl et al. investigated 38 revision arthroplasties, recording a 92% cumulative survival rate after a mean follow-up of 12 years [16]. In acetabular revision of 21 hips with substantial bone loss followed for 2–10 years, Symeonides et al. observed only 1 case of radiographic loosening and no mechanical failure filling bone cavities with autografts [43]. At 5-year follow-up, van Koeveringe and Ochsner found component migration in 9 of 33 hips (27%), in which the hip centre of rotation was not restored by autografts and APC, but no correlation between the extent of acetabular defects and cage migration [48]. Another few authors replaced bone loss using both autogenous and homogenous grafts. In combined acetabular lesions, Starker et al. [35] and Bonnomet et al. [37] revised 4 (2 aseptic and 2 septic)/43 and 5/21 loosened reconstructive cages at a mean of 5.8 and 8.75 years after surgery, respectively. A population of patients who received different designs of acetabular reinforcement systems, including 15 Burch-Schneider rings, and impaction autografting for combined bone stock defects and pelvic discontinuities showed an overall satisfactory outcome in 93% with no X-ray signs of loosening [34].

In another mixed series of 64 revisions (18 cases used an antiprotrusio device), Udomkiat et al. found an overall mechanical failure in 63.6% at a mean of 5.4 years. This was attributed to superior defects inadequately filled with bone chips [47]. Conversely, Haddad et al. reviewed 48 cases in which impacted grafts were protected by support rings (18 antiprotrusio cages), revealing excellent clinical and radiographic results at a mean of 64 months [46], and van der Linde and Tonino detected only 1 septic failure in a group of 16 revision THAs after a median of 10 years [17].

After an average follow-up period of 7.3 years, Winter et al. observed no cage loosening or migration and incorporation of the cancellous allograft into host bone in 38 cases. They concluded that a close fit between the graft and the acetabulum in addition to mechanical stability was crucial to their successful results [18]. Measuring migration with the EBRA analysis for a mean of 4.7 years, the same group of investigators followed 40 of 63 hips with severely damaged acetabula reconstructed with a Burch-Schneider cage and bone chips, assessing detectable migration in 30 cups [51]. Perka and Ludwig evaluated the outcome of a series of 63 revision hip arthroplasties using the APC, reporting only 3 aseptic and 2 septic loosenings and 2 additional radiographic failures at an average follow-up of 5.45 years. Segmental and combined bony defects were filled with either bulk (8 cases) or particulate allograft. The authors found a direct correlation between migration and posterior column defects and the increasing Paprosky stage. Indeed, all aseptic loosenings (3/12) occurred in patients with type IIIB defects and deficiency in the posterior column. These failures were independent of bulk or particulate allograft [44].

The use of solid bulk allografts and the Burch-Schneider cage in reconstructing severe bone loss was first reported by Gill et al. At an average follow-up of 8.5 years, 5 further revisions had been performed and, radiographically, 1 definitely loose, 2 probably loose, and 12 possibly loose reconstructions occurred in a group of 63 hips. They concluded that superior implantation leads to loosening, so the antiprotrusio cage in the absence of structural allograft is not recommended for significant posterior defects [28]. In a series of 103 acetabular revisions, Böhm and Banzhaf collected 26 cases using APC, with a success rate of 83% at a mean follow-up of 4.5 years, and positive outcomes were associated with bulk allografts (88/103) protected by a support ring [36]. Schatzker and Wong followed up for a mean of 6.6 years 38 patients who underwent revision with a Burch-Schneider cage and different bone grafts (autogenous or allogeneic, particulate or structural in only 6.7% of the cases), observing a failure rate of 5.4% [9]. At an average of 10.5 years, a 77% successful result was assessed by Saleh et al. in 9/12 hips treated with massive allografts and Müller or Burch-Schneider devices [15].

At a mean of 4.6 years, a stable reconstruction with no further acetabular operation and structural graft incorporation was detected by Goodman et al. in 32 of 42 hips (76%) with severe deficiencies, including 10 pelvic discontinuities, managed with the ilioischial Burch-Schneider ring. However, 3 revisions occurred because of recurrent dislocation, so overall mechanical failure was 84% [52]. Using a proper surgical technique to implant the cage and cancellous chips to fill the bone defects, Gallo et al. performed 69 revisions of acetabular bone loss classified as IIIA (32 cases) and IIIB (37 cases). At a mean follow-up of 8.3 years, 13 cages (18.8%) had been removed because of aseptic loosening (6), infection (5), and recurrent dislocation (2). Another 6 hips were radiographically definitely or probably loose, resulting in a 17.4% (12/69) rate of aseptic failure [19]. Pieringer et al. reported a survival rate of 93.4% at an average follow-up of 50.3 months, with cage explantation as the end point in a series of 67 Burch-Schneider rings implanted in primary and revision THA with no additional details concerning the type of graft and the severity of bone defects [49].

An unacceptable high rate of failure was observed by Paprosky et al., who revised severe acetabular deficiencies using ilioischial acetabular systems. This technique was successful in IIIA defects with 10-year survivorship of 78% but failed in 7/11 type IIIB hips, because sufficient host bone is not available to support a cage and allograft [53]. Boscainos et al. reported a 76% survivorship at 4.6 years with the use of a large structural graft supporting greater than 50% of the cup when protected by a cage (APC only in 2/72 cases). They attributed failures to the lack of a porous-coated surface providing biologic fixation [39].

Recently, Carroll et al., at a mean follow-up of 8.75 years, assessed successful outcome in 84% of 63 rings reconstructions of Paprosky III defects using morselized bone grafts and the Burch-Schneider cage in 55 cases (87.3%) [20]. In 2008, Sembrano and Cheng reviewed 72 cage reconstructions performed with the use of several devices (10 APCs) and different grafts for both cavitary and segmental defects (including pelvic discontinuity). Actuarial 5-year survivorships of 87.8% (cage removal), 80.7% (radiographic loosening), and 81.3% (any acetabular reoperation) were obtained. However, no single preoperative or intraoperative factor predicted cage failure [41].

Fifty-seven Burch-Schneider rings with the additional use of bone autografts were implanted by Symeonides et al. in 49 revision THAs for massive acetabular deficiency, observing a cumulative 10.5% failure rate, due to aseptic loosening in 2 cases and mechanical failure in 4 cases, at a mean of 11.5 years after operation [22]. Cuscujuela-Maña et al. reported 1 aseptic and 2 infected rerevisions in 91 hips with Paprosky IIC, IIIA, and IIIB defects at an average of 8.1 years. Radiographically, 3 additional cages were considered definitely loose [23]. In a single-surgeon consecutive series of 30 complex acetabular reconstructions using APC, Jones et al. observed 9-year survival of 95% and 92% for revision for any cause or further surgery as end points, respectively [24]. Finally, bulk allografts and the Burch-Schneider cage were effective in the management of 18 pelvic discontinuities and associated periprosthetic bone deficiency, with a cumulative 72.2% survival rate at 16.6 years [54].

Cage reconstruction is attractive for the possibility of spanning the acetabular defect, obtaining support from the ilium superiorly and the pubis and ischium inferiorly [15, 34]. Transferring load to the residual native bone, this mechanical bridge protects underlying bone graft from resorption, enabling osseous integration and restoring pelvic bone stock [9, 11, 12, 17].

Undoubtedly, defective primary stability is a negative factor for graft incorporation because excessive motion compromises the graft's ability to achieve bone healing, leading to failure of the reconstructive procedure. The use of the Burch-Schneider APC results in a mechanically adequate acetabular reconstruction, as demonstrated in an experimental study performed in fresh-frozen human pelves, where the antiprotrusio cage was stable under all cranial or dorsal defect conditions [55]. A simulation study using finite element analysis confirmed the ability of this device in reducing the stress distribution for the inner surface of the socket [56].

In the current series, the caudal fixation of the metal cage was obtained slotting the inferior flange into the ischium in 56 cases (86.1%). The remaining 9 revisions were performed positioning the ischial flange on the surface. Slotting the inferior flange into the ischium is technically demanding but provides a more stable anchorage of the cage reducing the risk of sciatic nerve injury [57].

Graft collapse with progressive migration of the prosthetic component is the major complication in acetabular revisions performed with massive allografts. However, the advantages of using a structural graft include immediate reconstitution of bone stock and restoration of the pelvic anatomy. The application of bulk allografts implanted in a protected environment with the addition of an antiprotrusio cage prevents early resorption and failure of the acetabular component.

Some authors found that cancellous allografts are too weak to support ring revisions [47, 51], therefore a structured allograft should be used for reconstruction of the cranial acetabular margin [11, 18, 28, 44, 52, 58]. Böhm and Banzhaf specifically investigated prognostic factors influencing survival of the cage, and the use of bulk allograft was identified to be able to provide a stable structural support in the early postoperative period [36].

Consequently, a close and tight fit between the graft and the residual acetabulum in addition to mechanical anchorage of the cage to the viable host bone is critical to the successful outcome of the reconstructive procedure [18, 21].

Polymethylmethacrylate is applied in the metal cavity to stabilize the polyethylene cup and should not be used to fix the cage to the bone. However, pressing cement in a doughy state during placement of the socket enables the penetration of a considerable amount of acrylic cement through the holes behind the APC. This cement, anchoring to the rough parts of the defective acetabular cavity and interdigitating with the bone graft, eventually increases the stability of the cup-cage system [11].

The major concern with standard acetabular cages is the lack of a porous coating for bone ingrowth. Consequently, the potential for biologic, long-term fixation through osseointegration is not predictable, and a high incidence of hardware failure due to screw breakage or ischial flange migration has been reported at midterm follow-up [42, 47]. However, most failures are likely related to a poor surgical technique and inadequate application of unsupportive bone grafts [11, 14]. At the time of our operations, the Burch-Schneider APC included a smooth titanium external surface, preventing osseous incorporation. As previously mentioned, we believe that the success of cage revision mainly depends on the initial stability of the reconstructed acetabulum and mechanical load protection of the structural graft, providing support to enhance bone healing. Once osseointegration has occurred, decreased load on the cage minimizes the risk of fatigue fracture compensating the inability for biologic fixation.

Undoubtedly, a porous or bioactive coating could enhance host bone response: to address the problem of unpredictable long-term durability, new generation acetabular cages (including the APC) are currently manufactured with potential for bone ingrowth [52, 59–61]. However, lower rates of rerevision or radiographic loosening have not been confirmed to date [58].

The breakage of a Burch-Schneider APC is a rarely encountered complication that is usually regarded as a mechanical failure induced by metal fatigue, facilitated by repetitive or excessive intraoperative recontouring of the flanges [44, 49, 52, 62]. We experienced only 1 case of late fracture in the transitional area to the proximal flange that occurred 13 years after the implantation in a young, male patient [63]. It was related to an accelerated polyethylene cup wear following metal-on-polyethylene coupling, which was promoted by the vertical placement of the cage. As it was the consequence of a delayed biological failure and not specifically of the reconstruction device, bone graft incorporation occurred as well. In over 200 cages implanted from 1992 to date, we observed no further cage breakage.

An additional limitation of the Burch-Schneider APC, as well as most ilioischial devices, is the need of an extensive surgical exposure of the ilium and ischium, as the placement of the cage is technically challenging. Uncommon but potentially devastating neurovascular injuries have been occasionally documented [52, 57, 62, 64–67].

Moreover, acetabular cages require prolonged delay in weight bearing to reduce excessive stress and prevent possible graft resorption and cage migration. Most failures may be detected on radiographs within two years after surgery, and the typical pattern of failure is disengagement of the ischial flange, reflecting the inability of the remaining bone to support the required mechanical loads. Authors who used conventional cages protecting their hips from weight bearing for 3 to 6 months have reported successful results even in massive defects [9, 12, 15, 18, 21, 22, 28]. Though the earlier availability of only two sizes of the Burch-Schneider cage prevented an optimal correspondence between the metal device and the grafted acetabulum in some cases, a great versatility was always experienced. Three additional sizes are currently available, facilitating the restoration of the centre of rotation of the hip to a near-anatomical position [5, 12, 14, 18, 46, 50]. The limitations of the present study include the retrospective review of the data, the relatively small number of patients, and the lack of a control group undergoing alternative techniques and different devices of reconstruction. However, to our knowledge, a comparable long-term outcome of acetabular revision using the same method of reconstruction, the Burch-Schneider cage and massive allograft, has never been reported previously. Moreover, all surgical procedures were performed in well-selected failed THAs, including the most severe bone defects.

## 5. Conclusions

Antiprotrusio cages have currently a limited but valuable role in the revision of the most complex cases of acetabular bone loss, including pelvic discontinuity [68]. Cages provide a large surface against the pelvis to span bone defects, distribute load, protect large bone grafts, and prevent early migration.

Acetabular reconstruction with the use of the Burch-Schneider antiprotrusio cage and bulk allografts has to be considered as a reliable procedure to manage severe periprosthetic deficiencies, enabling restoration of vital bone stock and providing highly successful long-term outcomes after revision arthroplasty.

Although only mid-term results are currently available, trabecular metal implants could provide an attractive alternative for complex acetabular revisions [69].
